# The impact of socioeconomic status on changes in cancer prevention behavior during the COVID-19 pandemic

**DOI:** 10.1371/journal.pone.0287730

**Published:** 2023-06-30

**Authors:** Mohamed I. Elsaid, Xiaochen Zhang, Anne L. R. Schuster, Jesse J. Plascak, Cecilia DeGraffinreid, Electra D. Paskett

**Affiliations:** 1 Department of Biomedical Informatics, College of Medicine, The Ohio State University, Columbus, Ohio, United States of America; 2 Secondary Data Core, Center for Biostatistics, College of Medicine, The Ohio State University, Columbus, Ohio, United States of America; 3 Division of Population Sciences, The Ohio State University Comprehensive Cancer Center, Columbus, Ohio, United States of America; 4 Department of Internal Medicine, Division of Cancer Prevention and Control, College of Medicine, The Ohio State University, Columbus, Ohio, United States of America; Wingate University, UNITED STATES

## Abstract

**Background:**

The impacts of socioeconomic status (SES) on COVID-19-related changes in cancer prevention behavior have not been thoroughly investigated. We conducted a cohort study to examine the effects of SES on changes in cancer prevention behaviors during the COVID-19 pandemic.

**Methods:**

We invited adult participants from previous studies conducted at Ohio State University to participate in a study assessing the impact of COVID-19 on various behaviors. Post-COVID-19 cancer prevention behaviors, including physical activity, daily intake of fruits and vegetables, alcohol and tobacco consumption, and qualitative changes in post-COVID-19 behaviors relative to pre-COVID levels, were used to construct a prevention behavior change index that captures the adherence status and COVID-related changes in each behavior, with higher index scores indicating desirable changes in prevention behaviors. Participants were classified into low, middle, or high SES based on household income, education, and employment status. Adjusted regression models were used to examine the effects of SES on changes in cancer prevention behaviors during the COVID-19 pandemic.

**Results:**

The study included 6,136 eligible participants. The average age was 57 years, 67% were women, 89% were non-Hispanic Whites, and 33% lived in non-metro counties. Relative to participants with high SES, those with low SES had a 24% [adjusted relative ratio, aRR = 0.76 (95%CI 0.72–0.80)], 11% [aRR = 0.89 (95%CI 0.86–0.92)], and 5% [aRR = 0.95 (95%CI 0.93–0.96)], lower desirable changes in prevention behaviors for physical activity, fruit and vegetable intake, and tobacco use, respectively. Low SES had a higher desirable change in alcohol consumption prevention behaviors, 16% [aRR = 1.16 (95%CI 1.13–1.19)] relative to high SES. The adjusted odds of an overall poor change in prevention behavior were adjusted odds ratio (aOR) 1.55 (95%CI 1.27 to 1.89) and aOR 1.40 (95%CI 1.19 to 1.66), respectively, higher for those with low and middle SES relative to those with high SES.

**Conclusion:**

The adverse impacts of COVID-19 on cancer prevention behaviors were seen most in those with lower SES. Public health efforts are currently needed to promote cancer prevention behaviors, especially amongst lower SES adults.

## 1. Introduction

The COVID-19 pandemic caused dramatic changes in activities of daily living. Since its onset, measures to control the pandemic have included quarantine orders, stay-at-home mandates, social distancing policies, and closures of schools and non-essential businesses [1]. Attributed to these changes, studies report shifts in individuals’ adherence to cancer prevention behaviors such as eating nutritious diets, consuming alcohol, and engaging in physical activity [2–5]. Yet, the pandemic [6] and its mitigation measures have inequitably harmed disadvantaged socioeconomic groups [7–9], with much less known about the differential impact of the pandemic on maintaining adherence to cancer prevention behaviors across the socioeconomic gradient.

Modifiable risk factors play a critical role in the development of cancer. Evidence indicates that cancer morbidity and mortality can be reduced by engaging in physical activity, consuming nutritious foods, and avoiding tobacco use and alcohol intake [10–15]. Accordingly, several organizations proposed recommendations to promote behaviors that, separately and in combination [16–18], could prevent new cancer diagnoses and deaths [19, 20]. These behaviors include quitting or never smoking, consuming alcohol in moderation (one or fewer drinks daily for women and two or fewer drinks daily for men), eating at least five non-starchy fruits and vegetables daily, and maintaining a weekly physical activity of at least 75 vigorous-intensity or 150 moderate-intensity minutes [10–15, 21, 22].

Adherence to cancer prevention behaviors is conditioned by multilevel factors that span the individual, social, economic, and physical environments [23]. Evidence indicates that socioeconomic status is inversely associated with adherence to cancer prevention behaviors. As such, individuals with a lower socioeconomic status show a higher prevalence of poor diet, physical inactivity, and tobacco use [24–26]. Alcohol consumption is higher among men of lower socioeconomic status [26, 27]. These differences reflect the fact that people with fewer resources experience more financial, structural, and personal obstacles to practicing cancer prevention behaviors [28].

As a result of COVID-19, multilevel factors known to influence the adherence to cancer prevention behaviors [29] have been altered [30–34] and could exacerbate existing disparities in the adherence to recommended cancer prevention behaviors. Our objective was to examine the impact of the COVID-19 pandemic on cancer prevention behaviors and the differential effects of socioeconomic status on changes in cancer prevention behavior throughout the pandemic. Understanding how the COVID-19 pandemic affected cancer prevention behaviors among people across the socioeconomic gradient is vital to inform cancer control strategies. Changes in cancer prevention behaviors at the population level can have long-term consequences in reducing cancer morbidity and mortality.

## 2. Materials and methods

This study was part of an NCI-funded initiative conducted in conjunction with 16 other NCI-designated Cancer Centers—the IC-4 (Impact of COVID-19 on the Cancer Continuum Consortium) (S1 Methods). The Institutional Review Board of The Ohio State University (OSU) approved this study in June 2020. We followed the Strengthening the Reporting of Observational Studies in Epidemiology (STROBE) reporting guidelines.

### 2.1. Theoretical framework

This study was based on the Health Belief Model (HBM) [35, 36]. According to the HBM, the change in health behaviors depends on a series of health beliefs, including 1) perceived susceptibility to exposure to COVID-19, 2) perceived severity of the consequences of contracting COVID-19 (e.g. hospitalization or death), 3) perceived benefits of the effectiveness of the proposed COVID-19 prevention measures, 4) perceived barriers to executing the proposed prevention measures, 5) cues to the proposed prevention actions, and 6) self-efficacy in the person’s ability to successfully perform COVID-19 prevention measures.

### 2.2. Setting

Participants from Ohio and Indiana who agreed to be re-contacted from previous studies conducted at the OSU Comprehensive Cancer Center (OSUCCC) were asked to participate in this study (S1 Methods). Ohio and Indiana mandated statewide stay-at-home orders on March 23 and March 24, 2020, respectively. Both states initiated a gradual reopening on 1 May 2020.

### 2.3. Sample selection

Eligible participants were adults 18 years or older who consented to participate in the study. To ensure the inclusion of the most vulnerable, underserved, and minority populations, we sought to recruit healthy adult volunteers, cancer patients, cancer survivors, and survivors’ caregivers in our catchment area in Ohio and Indiana. This was achieved by employing two recruitment strategies. First, we identified and reached out to individuals who had previously participated in OSU studies and consented to be contacted for future research projects. In addition, we invited all identified cancer patients and survivors to nominate their primary caregivers to participate in the study. Second, we utilized our community partners and the OSUCCC Pelotonia listservs to recruit participants to further enhance the representativeness of our study sample and ensure the inclusion of minority and underserved communities.

### 2.4. Data collection

We used several data collection methods, including web, telephone, and mailed surveys. Respondents with valid emails received an initial survey invitation email and three reminders seven days apart. All participants were initially selected using an eligibility form to confirm their current Ohio or Indiana residence before conducting the survey. Participants were able to save the web survey and resume it at a later time. Those who partially completed the web survey received an email reminder one week after they had last accessed the survey. A trained interviewer contacted participants without an email address and those with invalid emails on file by phone. Participants who were initially reached by phone were offered the option to complete the survey over the phone or online. For those who requested a mailed survey, we sent a cover letter and a paper survey with a self-addressed stamped return envelope. For non-English-speaking participants, a bilingual staff member administered the survey in the appropriate language. Participants were offered a $10 gift card upon completion of the survey. Data were collected and managed using the Research Electronic Data Capture (REDCap) secure web-based application hosted at OSU. Data were collected from June 19, 2020, through November 30, 2020.

### 2.5. Survey development

The survey elements (S1 Table) were finalized in conjunction with other members of the IC-4 [37]. The survey included individual behaviors related to mitigation of COVID-19 transmission, challenges related to social distancing, self/family isolation, stress, and health behaviors highly relevant to cancer and other chronic diseases. Questions also assessed perceived stigma associated with COVID-19 with respect to different population groups and covariates, such as health literacy and mental health, suspected of moderating these influences.

### 2.6. Study measures

#### 2.6.1. Exposure

The primary exposure was individual-level SES. Respondents were asked to report their highest grade or level of school completed, their combined annual household income, and their current employment status (S2 Table). We used the responses to these three questions to develop an aggregate SES score. The participants’ responses to questions about total household income and highest attained education level were scored between 0 and 3 and between 0 and 2 for the self-reported employment status. The aggregate SES score was then used to classify participants into one of three groups: low SES [lower quartile (SES scores 0 to 4)], middle SES (SES scores 5 and 6), or high SES [upper quartile (SES scores 7 and 8)].

#### 2.6.2. Outcomes

Our primary outcome was the post-COVID-19 change in cancer prevention behaviors relative to pre-COVID-19 levels. Participants were asked to report current (i.e., post-COVID-19 pandemic) cancer prevention behaviors, including the number of days per week of physical activity of at least moderate intensity, the total daily frequency of fruit and vegetable intake, alcohol consumption, the number of binge drinking days of five or more alcoholic beverages on the same occasion, and tobacco use. We classified participants as adherent and non-adherent on each post-COVID-19 cancer prevention behavior using established cancer prevention recommendations [19, 20]. Additionally, participants described qualitative changes in each of the self-reported post-COVID-19 cancer behaviors relative to the pre-COVID-19 levels (i.e., same, more, or less]. We combined participants’ adherence statuses on each prevention behavior with the post-COVID-19 behavior changes to construct a two-dimensional 6-point cancer prevention behavior change (CPBC) score for each of the four cancer prevention behaviors (i.e., 24-point CPBC score). The resulting score captured the adherence status and COVID-related changes in each behavior, with higher CPBC scores indicating desirable changes in prevention behavior. The aggregate CPBC score was used to rank participants’ prevention behavior changes during the COVID-19 pandemic into one of four quartiles: poor (0 to 13), average (14 to 15), good (16 to 18), and excellent (≥18) (S3 Table).

### 2.7. Statistical analysis

Overall and stratified characteristics were summarized using descriptive statistics, including means and standard deviations (SD) for continuous variables and frequencies and proportions for categorical variables. Differences between participants with low, middle, and high SES were compared using analysis of variance (ANOVA) for continuous variables and χ^2^ or Fisher’s exact tests for categorical variables. We used modified Poisson regression models [38] to examine the adjusted associations, on the relative ratios (aRR) scale, between SES and each of the cancer prevention behaviors, using the 6-point CPBC score. We constructed adjusted multinomial logistic regression models with excellent aggregate cancer prevention behavior change as the reference outcome to assess the association between SES and COVID-19 related changes in cancer prevention behaviors. All statistical analyzes were conducted using SAS v9.4, with two-tail tests and a significance level of 0.05.

### 2.8. Sensitivity analysis

In a sensitivity analysis, all missing values were imputed using multiple imputations by chained equations to create 10 imputed data sets [39]. The imputation by chained equations approach utilizes a flexible variable-by-variable multivariable imputation model to address missing data for datasets with complex data structures. As such, we used logistic regression-based imputation models to impute binary and ordinal variables, the discriminant function to impute nominal variables, and a regression-based approach with predictive mean matching to impute continuous variables. The parameter estimates obtained from each imputed data set were combined using the Rubin method [40].

## 3. Results

**Fig 1:**
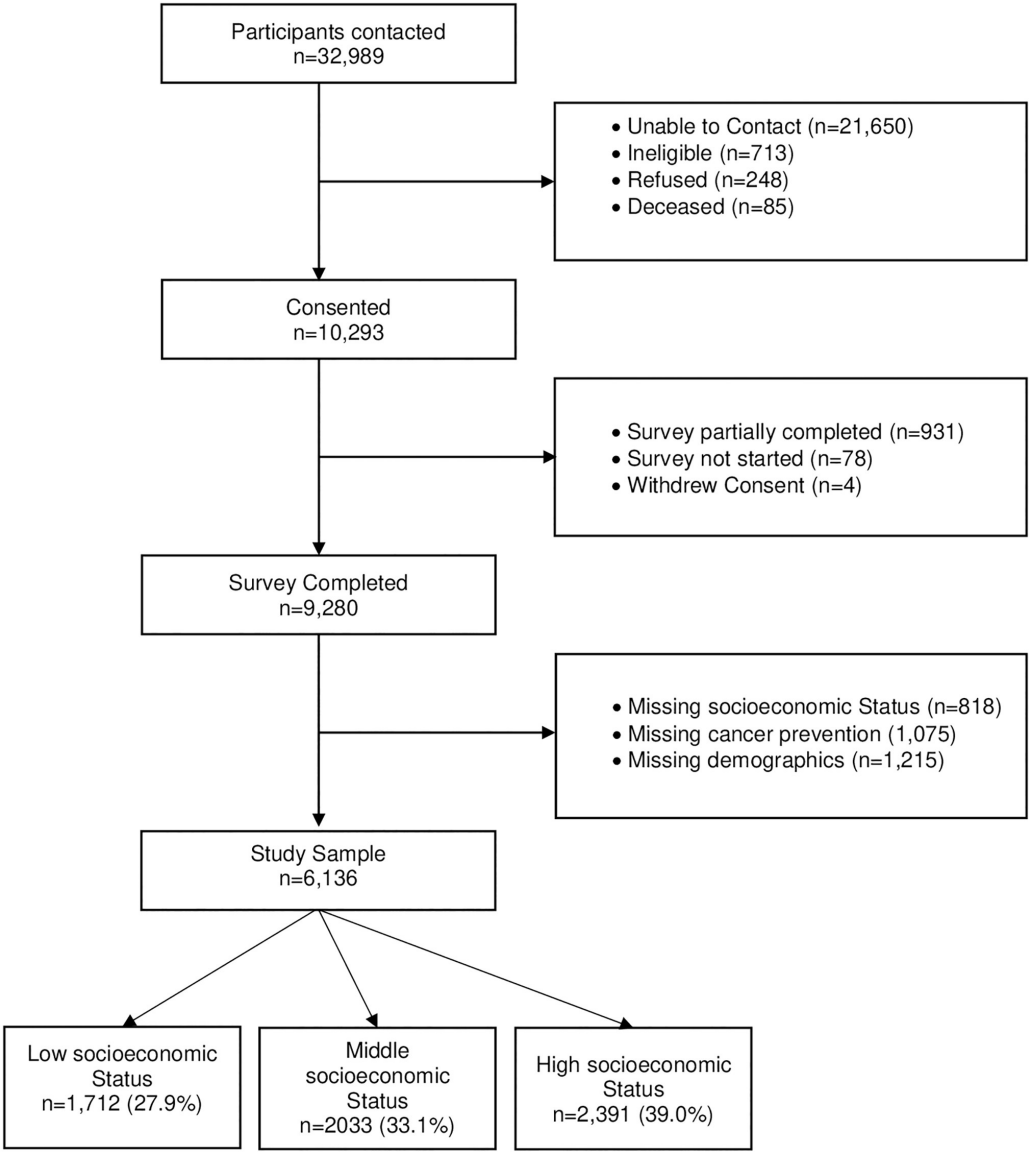
Study schema.

The study sample included 6,136 eligible participants (Fig 1). The overall and SES stratified characteristics of the survey participants are described in Table 1. The sample mean age (SD) was 56.8 (13.2) years, 67.1% were female, 88.6% were non-Hispanic White and 32.5% lived in non-metro counties. An estimated 75% of the participants were married or lived as married and 57.5% were enrolled in private insurance. Higher SES was significantly associated with younger age, higher probability of being a male, identified as White non-Hispanic, being married/living as married, having private insurance, and residing in a non-rural county.

**Table 1:** Characteristics of survey participants by socioeconomic status (n = 6,136).

Demographics	All (n = 6,136)	Socioeconomic Status*			P-value
		Low (n = 1,712)	Middle (n = 2,033)	High (n = 2,391)	
**All (%)**					
**Age,** years					< .0001^§^
Mean (SD)	56.8 (13.2)	58.3 (14.3)	57.6 (13.0)	54.9 (12.4)	
Median (25^th^, 75^th^)	58.0 (48.0 to 67.0)	61.0 (49.0, 69.0)	59.0 (50.0, 67.0)	55.0 (47.0, 64.0)	
**Age,** years					< .0001^†^
18–34	452 (7.4)	156 (9.1)	131 (6.4)	165 (6.9)	
35–49	1217 (19.8)	273 (16)	351 (17.3)	593 (24.8)	
50–64	2543 (41.4)	603 (35.2)	870 (42.8)	1070 (44.8)	
65+	1924 (31.4)	680 (39.7)	681 (33.5)	563 (23.6)	
**Sex**					0.003^†^
Male	2018 (32.9)	494 (28.9)	676 (33.3)	848 (35.5)	
Female	4118 (67.1)	1218 (71.1)	1357 (66.8)	1543 (64.5)	
**Race Ethnicity**					< .0001^†^
White, Non-Hispanic	5434 (88.6)	1438 (84)	1811 (89.1)	2185 (91.4)	
Black, Non-Hispanic	326 (5.3)	151 (8.8)	104 (5.1)	71 (3)	
Hispanic	127 (2.1)	41 (2.4)	44 (2.2)	42 (1.8)	
Other^‡^	249 (4.1)	82 (4.8)	74 (3.6)	93 (3.9)	
**Marital Status**					< .0001^†^
Single, Never Married	556 (9.0)	237 (13.8)	173 (8.5)	146 (6.1)	
Married/Living as Married	4604 (75.0)	986 (57.6)	1597 (78.6)	2021 (84.5)	
Widowed, Separated or Divorced	976 (15.9)	489 (28.6)	263 (12.9)	224 (9.4)	
**Health Insurance**					< .0001^†^
None	182 (3.0)	106 (6.2)	63 (3.1)	13 (0.5)	
Public Insurance	795 (13.0)	507 (29.6)	200 (9.8)	88 (3.7)	
Private Insurance	3528 (57.5)	500 (29.2)	1191 (58.6)	1837 (76.8)	
Public & Private Insurance	1631 (26.6)	599 (35.0)	579 (28.5)	453 (19.0)	
**State**					< .0001^†^
Indiana	233 (3.8)	94 (42.3)	68 (35.0)	71 (23.2)	
Ohio	4145 (96.2)	1618 (57.7)	1965 (65.0)	2320 (76.8)	
**Region of Residence**					< .0001^†^
Rural	1991 (32.5)	724 (42.3)	712 (35.0)	555 (23.2)	
Metro	4145 (67.6)	988 (57.7)	1321 (65.0)	1836 (76.8)	

* Including education, household income, and occupational status

^†^ Chi-Square tests from the association between each demographic variable and socioeconomic status

^‡^ Including participants who self-identified with more than one racial group

^§^ One-way ANOVA

The impacts of the COVID-19 pandemic on cancer prevention behaviors are outlined in Table 2. An estimated 42.3% of participants were physically inactive or had less physical activity post-COVID-19. Less fruit and vegetable intake post-COVID-19 was reported by 10.5% of participants. Alcohol consumption increased post the COVID-19 pandemic in 15.2% of participants with 21.2% of those who reported alcohol consumption pre-COVID-19 reporting binge drinking for at least one day in the past 30 days. More tobacco use post-COVID-19 was reported by 1.5% of participants.

**Table 2:** Cancer prevention behavior changes post the COVID-19 pandemic by socioeconomic status (n = 6,136).

Cancer Prevention Behavior^‡^	All (n = 6,136) % (95% CI)	Socioeconomic Status*			P-value^†^
		Low (n = 1,712)	Middle (n = 2,033)	High (n = 2,391)	
		% (95% CI)	% (95% CI)	% (95% CI)	
**Cancer Prevention Behavior Change** ^§^					< .0001
Poor	29.8 (28.6–30.9)	31.8 (29.6–34.1)	30.6 (28.6–32.6)	27.6 (25.8–29.4)	
Average	23.8 (22.8–24.9)	26.9 (24.8–29.0)	23.6 (21.7–25.5)	21.9 (20.3–23.6)	
Good	22.0 (21.0–23.0)	20.0 (18.1–22.0)	22.5 (20.7–24.4)	23.0 (21.3–24.7)	
Excellent	24.4 (23.3–25.5)	21.3 (19.4–23.3)	23.4 (21.5–25.3)	27.5 (25.7–29.4)	
**Physical Activity**					< .0001
Not Physical Activity	14.0 (13.2–14.9)	22.9 (20.9–25.0)	13.3 (11.9–14.9)	8.2 (7.2–9.4)	
Less Physical Activity	28.3 (27.1–29.4)	27.3 (25.2–29.5)	29.2 (27.3–31.3)	28.1 (26.3–30.0)	
Same Physical Activity	38.0 (36.8–39.2)	37.7 (35.4–40.0)	40.4 (38.2–42.6)	36.1 (34.2–38.1)	
More Physically Active	19.8 (18.8–20.8)	12.1 (10.6–13.7)	17.1 (15.5–18.8)	27.5 (25.7–29.4)	
**Fruit and Vegetable Intake**					< .0001
No Fruit or Vegetable Intake	3.7 (3.2–4.2)	5.7 (4.6–6.9)	3.5 (2.7–4.4)	2.4 (1.8–3.1)	
Less Intake	10.5 (9.7–11.3)	12.9 (11.4–14.6)	10.3 (9.0–11.7)	8.8 (7.7–10.0)	
Same Intake	73.2 (72.0–74.3)	69.2 (66.9–71.3)	74.9 (73.0–76.8)	74.5 (72.7–76.3)	
More Intake	12.7 (11.9–13.6)	12.3 (10.8–13.9)	11.3 (9.9–12.7)	14.3 (12.9–15.7)	
**Alcohol Consumption**					< .0001
No Alcohol Intake	38.8 (37.6–40.0)	57.6 (55.2–60.0)	36.8 (34.7–38.9)	27.1 (25.3–28.9)	
Less Intake	6.4 (5.8–7.1)	4.3 (3.4–5.3)	7.6 (6.5–8.8)	6.9 (6.0–8.0)	
Same Intake	39.6 (38.3–40.8)	30.1 (27.9–32.3)	42.6 (40.4–44.7)	43.8 (41.8–45.9)	
More Intake	15.2 (14.3–16.2)	8.1 (6.8–9.5)	13.1 (11.7–14.6)	22.2 (20.5–23.9)	
**Binge Alcohol Drinking** ^||^					0.586
Yes	21.4 (20.1–22.7)	21.1 (18.2–24.2)	22.3 (20.1–24.7)	20.8 (18.9–22.8)	
No	78.6 (77.3–79.9)	78.9 (75.8–81.8)	77.7 (75.3–79.3)	79.2 (77.2–81.1)	
**Tobacco Use**					< .0001
No Tobacco Use	89.6 (88.8–90.3)	82.8 (80.9–84.5)	90.4 (89.0–91.7)	93.8 (92.7–94.7)	
Less Intake	1.5 (1.2–1.8)	3.0 (2.3–4.0)	1.3 (0.8–1.9)	0.5 (0.2–0.8)	
Same Intake	6.0 (5.4–6.6)	9.1 (7.8–10.6)	5.4 (4.5–6.5)	4.1 (3.4–5.0)	
More Intake	1.5 (1.2–1.8)	5.1 (4.1–6.2)	2.9 (2.2–3.7)	1.6 (1.2–2.2)	

* Included measures for education, household income, and occupational status

^†^ Chi-Square Tests from the association between each cancer prevention behavior and socioeconomic status

^‡^ Compared to the pre- COVID-19 pandemic levels

^§^ Classification is based on the quartiles of a 24-point post-COVID-19 cancer prevention behavior change score that included physical activity (0–6 points), fruit and vegetable intake (0–6 points), alcohol consumption (0–6 points), and tobacco use (0–6 points). A higher score quartile indicates better prevention behavior change. Score quartiles cutoff points were poor (0 to 13), average (14 to 15), good (16 to 18), and excellent (≥18)

^||^At least one day during the past 30 days with 5 or more alcoholic drinks _on the same occasion amongst participants with alcohol consumption (n = 3,755)

CI = Confidence Interval

Cancer prevention behavior changes post the COVID-19 pandemic were significantly associated with SES (Table 2). The proportion of participants in the excellent cancer prevention behavior quartile increased significantly with higher SES [low SES vs. high SES; 21.3% vs. 27.5%; P-value < .001]. Relative to pre-COVID-19 levels, higher SES was significantly associated with increases in the post-COVID-19 prevalence of more physical activity [low SES vs. high SES; 12.1% vs. 27.5%; P-value < .001], higher fruit and vegetable intake [low SES vs. high SES; 12.3% vs. 14.3%; P-value < .001], and more alcohol consumption [low SES vs. high SES; 8.1% vs. 22.2%; P-value < .001]. Higher SES was also associated with lower tobacco use [low SES vs. high SES; 3.0% vs. 0.5%; P-value < .001].

**Fig 2:**
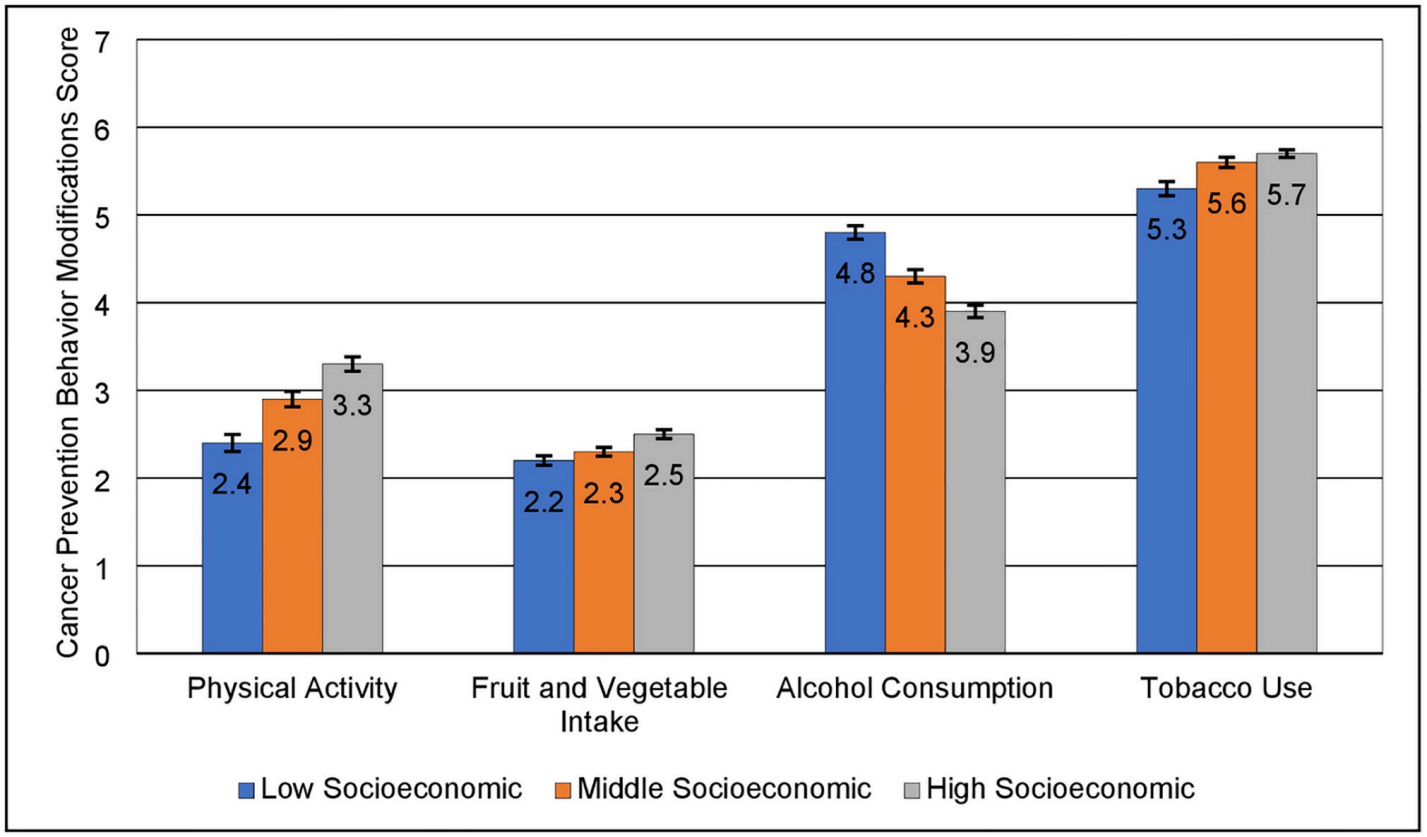
Mean post COVID-19 cancer prevention behavior change scores by socioeconomic status. The score includes measures of physical activity (0–6), fruit and vegetable intake (0–6), alcohol consumption (0–6), and tobacco use (0–6) compared to levels before the COVID-19 Pandemic. A higher score indicates better prevention behavior.

The average CPBC score increased with higher SES for physical activity, fruit and vegetable intake, and tobacco use, indicating higher desirable cancer prevention behavior changes post-COVID-19 among participants with higher SES (Fig 2). However, the mean CPBC score for alcohol consumption was higher in the low vs. high SES (4.8 vs. 3.9), indicating lower desirable post-COVID-19 behavior changes among participants in the high SES. Table 3 describes the adjusted RR for the effects of SES on the CPBC score for each cancer prevention behavior. Relative to participants with high SES, those with low SES had a 24% [aRR = 0.76 (95% CI: 0.72–0.80)], 11% [aRR = 0.89 (95% CI: 0.86–0.92)], and 5% [aRR = 0.95 (95% CI: 0.93–0.96)], lower CPBC score for physical activity, fruit and vegetable intake, and tobacco use, respectively. However, low SES was associated with a better CPBC score for alcohol consumption, 16% [aRR = 1.16 (95% CI: 1.13–1.19)] relative to high SES. Similar trends were observed for the middle vs. high SES associations (Table 3).

**Table 3:** Adjusted relative ratios (aRR) for factors associated with higher* cancer prevention behavior change score post the COVID-19 pandemic (n = 6,136).

Factor	More Physical Activity	More Fruit & Vegetable Intake	Less Alcohol Consumption	Less Tobacco Use
	aRR (95% CI)	aRR (95% CI)	aRR (95% CI)	aRR (95% CI)
**Age,** years				
18–34	Ref.	Ref.	Ref.	Ref.
35–49	0.94 (0.87–1.01)	0.98 (0.92–1.04)	**1.10 (1.03–1.17)**	1.00 (0.96–1.04)
50–64	0.96 (0.89–1.03)	0.99 (0.94–1.05)	**1.23 (1.16–1.30)**	**1.04 (1.00–1.07)**
65+	1.02 (0.93–1.11)	1.03 (0.96–1.10)	**1.26 (1.18–1.34)**	**1.16 (1.11–1.20)**
**Sex**				
Male	Ref.	Ref.	Ref.	Ref.
Female	0.98 (0.94–1.01)	**1.11 (1.08–1.14)**	**1.05 (1.03–1.08)**	**1.06 (1.04–1.08)**
**Race Ethnicity**				
White, non-Hispanic	Ref.	Ref.	Ref.	Ref.
Black, non-Hispanic	**0.85 (0.77–0.93)**	1.04 (0.98–1.11)	1.04 (1.00–1.09)	1.01 (0.97–1.04)
Hispanic	1.08 (0.96–1.21)	**1.10 (1.00–1.21)**	1.04 (0.96–1.12)	1.02 (0.97–1.07)
Other^†^	1.04 (0.95–1.14)	**1.11 (1.03–1.19)**	**1.14 (1.08–1.19)**	1.03 (0.99–1.06)
**Marital status**				
Single, Never Married	Ref.	Ref.	Ref.	Ref.
Married/Living as Married	0.99 (0.93–1.06)	1.00 (0.95–1.06)	**0.95 (0.91–0.99)**	1.03 (1.00–1.06)
Widowed, Separated or Divorced	0.98 (0.90–1.06)	0.96 (0.90–1.02)	**0.94 (0.90–0.98)**	1.01 (0.98–1.04)
**Health Insurance**				
Public & Private Insurance	Ref.	Ref.	Ref.	Ref.
None	1.11 (0.98–1.27)	1.03 (0.93–1.14)	1.07 (1.00–1.14)	0.97 (0.91–1.03)
Public Insurance	0.97 (0.90–1.05)	0.97 (0.93–1.02)	1.00 (0.97–1.04)	0.96 (0.94–0.99)
Private Insurance	**1.09 (1.02–1.17)**	1.02 (0.97–1.07)	**0.93 (0.89–0.96)**	**1.07 (1.04–1.10)**
**State**				
Ohio	Ref.	Ref.	Ref.	Ref.
Indiana	0.96 (0.87–1.07)	0.99 (0.92–1.07)	1.02 (0.97–1.07)	1.01 (0.98–1.04)
**Region of Residence**				
Metro	Ref.	Ref.	Ref.	Ref.
Rural	1.00 (0.96–1.05)	0.97 (0.94–1.00)	**1.02 (1.00–1.05)**	0.99 (0.98–1.01)
**Socioeconomic Status** ^‡^				
High	Ref.	Ref.	Ref.	Ref.
Middle	**0.89 (0.85–0.92)**	**0.92 (0.89–0.94)**	**1.05 (1.02–1.07)**	**0.98 (0.97–0.99)**
Low	**0.76 (0.72–0.80)**	**0.89 (0.86–0.92)**	**1.16 (1.13–1.19)**	**0.95 (0.93–0.96)**

* Passion regression with robust error variance with each 6-point cancer prevention behavior change score as outcome adjusted for age, sex, race-ethnicity, marital status, state, region of residence, health insurance, and socioeconomic status. A higher score indicates better change in cancer prevention behavior post the COVID-19 pandemic

^†^ Including participants who self-identified with more than one racial group

^‡^ Included measures for education, household income, and occupational status; RR = Relative Ratio; CI = Confidence Interval

**Fig 3:**
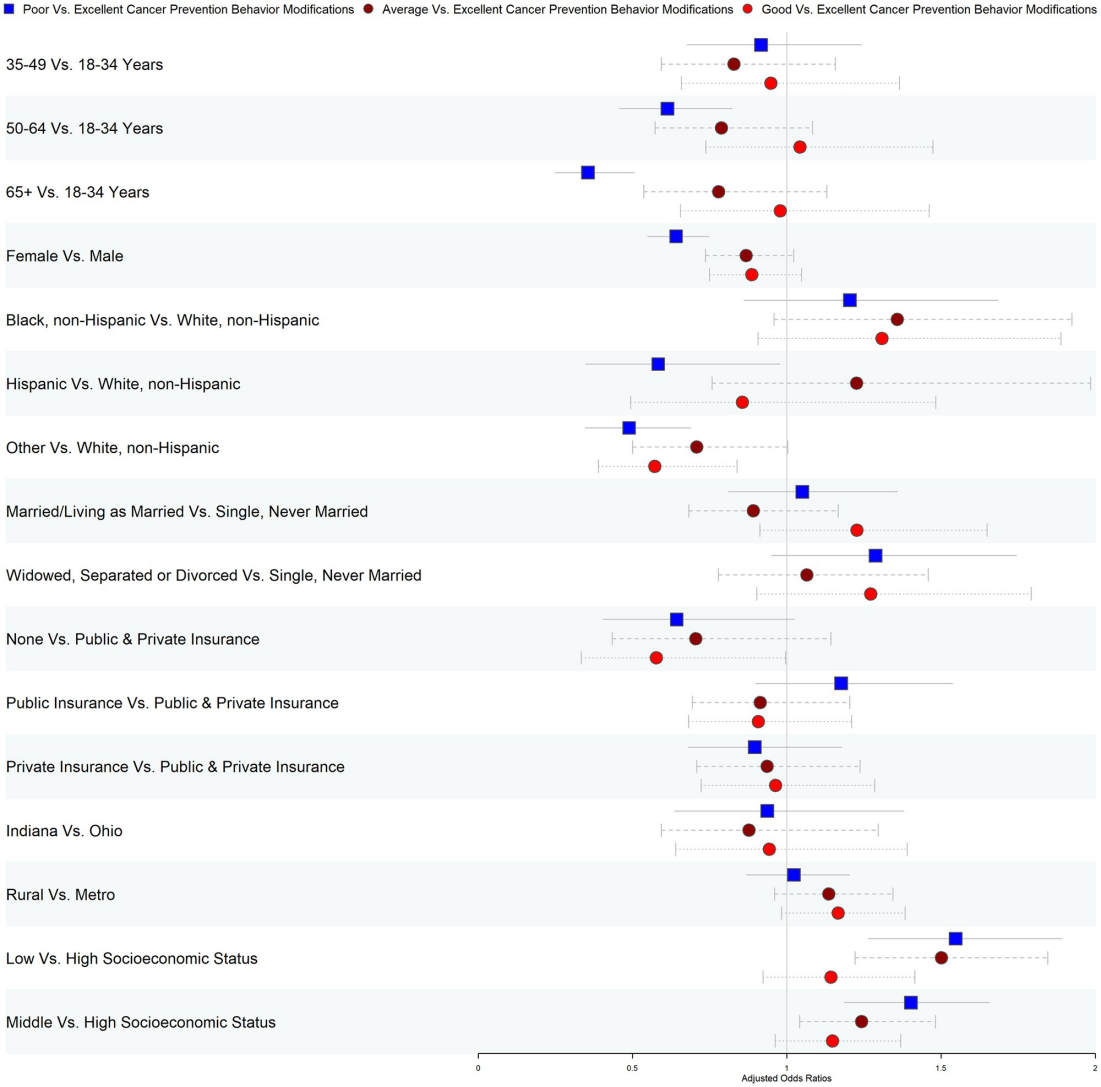
Adjusted* odds ratios for factors associated with overall cancer prevention behavior changes post the COVID-19 pandemic (n = 6,136). *Multinomial logistic regression models adjusting for age, sex, race-ethnicity, marital status, state, region of residence, health insurance, and socioeconomic status.

Fig 3 and S4 Table denotes the effects of SES on the overall cancer prevention behavior changes post the COVID-19 pandemic. Relative to participants with excellent CPBC post-COVID-19, the adjusted odds of poor CPBC for low SES were adjusted odds ratio (aOR) 1.55 (95% CI: 1.27–1.89) and aOR 1.40 (95% CI: 1.19–1.66) for middle SES, respectively compared with high SES. Compared to those with high SES, low and middle SES participants had higher odds of average CPBC relative to excellent CPBC. However, SES status was not a significant predictor for the good vs. excellent CPBC association. Results from the sensitivity analysis using multiple imputations were consistent with our main findings (S5 and S6 Tables).

## 4. Discussion

This study is novel in its investigation of individuals’ adherence to cancer-prevention behaviors during the COVID-19 pandemic in a sample of adults from Ohio and Indiana. Our findings reveal a differential impact of COVID-19 on the adherence to cancer prevention behaviors according to socioeconomic status, exacerbating existing inequities. The adverse impacts of COVID-19 on cancer prevention behaviors were seen primarily in those with a lower socioeconomic status. Compared to pre-COVID levels, individuals with low SES were more likely to report less physical activity, less fruits and vegetables consumption, and increased tobacco use. On the contrary, individuals with higher socioeconomic status were more likely to report even better levels of cancer prevention behaviors than before the pandemic, with the exception to alcohol consumption.

Our study builds on the limited but growing literature on the differential impacts of the COVID-19 pandemic on health-promoting behaviors across socioeconomic groups. Consistent with our findings, previous studies on the early effects of the pandemic’s lockdowns on physical activity reported widening inequalities in physical activity levels between those of low and high socioeconomic status [3, 41]. In contrast, another study that evaluated the pandemic’s disruption on a broader array of healthy behaviors (sleep, diet quality, physical activity, frequency of alcohol consumption, and frequency of snacking), observed both positive and negative changes in lifestyle behaviors, irrespective of socioeconomic status [2].

A broad literature explores how socioeconomic status shapes motivations for healthy behavior and offers insights into why we observed that COVID-19 exacerbated socioeconomic disparities in adherence to cancer prevention behaviors [28]. Motives for healthy behavior focus on stress, perceived lower benefits, class distinctions, and knowledge of risk. The literature suggests that higher socioeconomic groups face less stress, where stress might encourage coping through unhealthy behavior, and gain more longevity benefits from healthy behavior. Additionally, higher socioeconomic groups accrue prestige by setting themselves apart with healthy behavior, and by adopting healthier behaviors because they have greater knowledge of the risks of unhealthy behavior [28]. Given the relevance to COVID-19, we consider the role that stress may play as well as and the perceived benefit of healthy behaviors.

Related to stress, in 2020, low-wage workers lost jobs at five times the rate of middle-wage workers [7], and people who lost their jobs were more likely to experience material and financial hardships and food insecurity [9, 42, 43]. Low-income adults were nearly twice as likely to report major negative mental health impacts, such as serious psychological distress [44], as compared to high-income adults [8]. This could have unequal implications on coping behaviors and addiction to substances like alcohol and tobacco [45–47]. Though it is interesting to note that financial crises have been associated with reduced substance use overall, possibly because individuals have less money to spend on harmful substances [48, 49].

The limited or uncertain benefit of adhering to cancer prevention behaviors could alternatively explain our results. Throughout the pandemic, people of lower socioeconomic status were more likely to work in essential jobs that did not allow them to work from home. This includes but is not limited to warehouse and grocery workers, bus drivers, and those in other forms of public transport. Based on the literature [28], perhaps their increased exposure to the virus reduced the perceived benefits of adhering to cancer prevention behaviors.

While individual motives and behaviors may be influenced by socioeconomic status, addressing disparities in cancer prevention behaviors requires a multi-level approach. As the effects of COVID-19 continue to unfold, ongoing research and multi-level strategies are needed to promote cancer prevention behaviors equitably. Expanding health insurance coverage may be one such strategy, where evidence shows that Medicaid and Medicaid expansion is linked to increased financial security and reduced poverty rates [50–53]. The COVID-19 pandemic also spurred new funding and programs to address health-related social needs. Researchers have and could continue to evaluate the impact of policy changes made during COVID-19 to identify strategies for reducing disparities in cancer prevention behaviors. Preliminary evidence suggests that value-based payments may be a potential way to pay for services that could support cancer prevention behaviors, including food supports [54]. Similarly, hospitals and health systems could increase their use of patient navigators or adopt cross-sector collaboration software to optimize referrals to community resources. Access to these technologies would enable clinicians to refer their patients to local community resources that could help them overcome barriers to cancer prevention behaviors. Finally, as noted by many others, it is critical to engage individuals and communities who have historically been excluded from policy design and implementation to ensure solutions reflect their priorities [54].

This study had multiple strengths. First, the survey followed a long-standing and well-established theoretical framework and included factors known to impact health disparities. Second, the survey assessed a range of recommended cancer prevention behaviors and included several measures of socioeconomic status. Finally, our novel CPBC score improves upon existing methods for measuring adherence to cancer prevention behaviors. Unlike previous measures, the CPBC score is two-dimensional and accounts for adherence and behavior changes over time, which is essential when considering long-term risk of developing cancer. Also, previous measures inadequately account for behaviors associated with the major risk factors of cancer, especially tobacco use.

Despite these strengths, we note several limitations. One limitation is the self-reported nature of the data collected, which introduces the potential of recall, social desirability, and selection bias. While not atypical for responses to survey questions about income, selection bias may be a concern because approximately one-third of participants were excluded from the main analysis of our study because they did not report household income or other study measures. However, the results from our sensitivity analysis using multiple imputations, which were consistent with our main findings, provide strong support against this concern. In addition, our study sample was not representative of the state of Ohio. Also, data collection spanned a six-month period in which the context of COVID surges and responses were not uniform and could have differentially impacted cancer prevention behaviors reported by participants. Yet, in collecting data beyond the pandemic’s early months, our study provides important, and timely insights into the longer-term impacts of the pandemic.

The current findings provide crucial insights about individuals at heightened risk during the pandemic. These results underscore the importance of formally recognizing and addressing the root causes of disparities in cancer prevention behaviors, including economic inequities, through targeted multi-level intervention strategies. It is imperative that we take action to address these disparities and ensure that all individuals have access to the resources and support they need to prevent cancer and maintain their health.

## Supporting information

S1 Methods Study sitting. (DOCX)

S1 Table Survey elements by core constructs. (DOCX)

S2 Table Questions used to construct the aggregate socioeconomic status measure*. * The aggregate score was used to classify participants into low socioeconomic status (SES scores 0 to 4), middle socioeconomic status (SES scores 5 and 6), or high socioeconomic status (SES scores 7 and 8). †Including unemployed, students, homemaker, or disabled.
(DOCX)

S3 Table Definition of the cancer prevention behavior modification (CPBM) score. (DOCX)

S4 Table Adjusted odds ratios for factors associated with overall cancer prevention behavior changes post the COVID-19 pandemic (n = 6,136). Multinomial logistic regression models adjusting for age, sex, race-ethnicity, marital status, state, region of residence, health insurance, and socioeconomic status. * Included post-COVID-19 measures of physical activity, fruit and vegetable intake, alcohol consumption, and tobacco use compared to levels before the COVID-19 Pandemic. † Including participants who self-identified with more than one racial group. ‡ Included measures for education, household income, and occupational status. OR = Odds Ratio; CI = Confidence Interval.
(DOCX)

S5 Table Adjusted relative ratios (aRR) for factors associated with higher* cancer prevention behavior modification score post the COVID-19 pandemic using multiple imputations (n = 9,280). All missing values were imputed using multiple imputations by chained equations to create ten imputed data sets. The parameter estimates obtained from each imputed data set were combined using the Rubin Method. * Passion regression with robust error variance with each 6-point cancer prevention behavior modification score as outcome adjusted for age, sex, race-ethnicity, marital status, region of residence, health insurance, and socioeconomic status. A higher score indicates better cancer prevention behavior modification post the COVID-19 Pandemic. † Including participants who self-identified with more than one racial group. ‡ Included measures for education, household income, and occupational status. RR = Relative Ratio; CI = Confidence Interval.
(DOCX)

S6 Table Adjusted odds ratios for factors associated with overall cancer prevention behavior modifications post the COVID-19 pandemic using multiple imputations (n = 9,280). All missing values were imputed using multiple imputations by chained equations to create ten imputed data sets. The parameter estimates obtained from each imputed data set were combined using the Rubin Method. Multinomial logistic regression models adjusting for age, sex, race-ethnicity, marital status, region of residence, health insurance, and socioeconomic status. * Include post-COVID-19 measures of physical activity, fruit and vegetable intake, alcohol consumption, and tobacco use compared to levels before the COVID-19 Pandemic. † Including participants who self-identified with more than one racial group. ‡ Included measures for education, household income, and occupational status. OR = Odds Ratio; CI = Confidence Interval.
(DOCX)
