# Supplementary material for: The impact of socioeconomic status on changes in cancer prevention behavior during the COVID-19 pandemic
Source: PLoS One. 2023 Jun 30;18(6):e0287730. doi: 10.1371/journal.pone.0287730 (PMC10313075; doi:10.1371/journal.pone.0287730)
Supplement: S1 Methods — (DOCX) [file pone.0287730.s001.docx]

**Supplemental Online Content**

**Supplementary Methods. Study Sitting**

This study was part of the NCI-funded IC-4 (Impact of COVID-19 on the Cancer Continuum Consortium). The initiative was funded to work collectively to develop core survey items and implement population surveys in each cancer center catchment area. The overall goal of the IC-4 was to assess how demographic differences impact engagement in cancer-preventive behaviors and cancer management/survivorship behaviors in the context of the COVID-19 environmental constraints. Each site had its theoretical framework and survey methods. Our site used the IC-4 core set of common data elements, augmented with issues specific to Ohio, with remote data collection methods to include many unique and diverse populations.

Ohio has many diverse populations, including 50 of 88 counties designated as rural, and 32 counties are part of the Appalachian area of the US. The Indiana participants were mainly from rural counties due to the eligibility of the respective prior study they had participated in. Rural and Appalachian areas are healthcare professional shortage areas, with poor access to the internet and cell phone coverage – limiting the availability of reliable and accurate information about the impacts of COVID-19. Ohio urban areas are sociodemographically diverse with respect to self-reported race-ethnicity, both established and new immigrants – many of the latter do not speak English and have relied on the sociality of their community for support and information in the past.
