# Supplementary material for: The impact of socioeconomic status on changes in cancer prevention behavior during the COVID-19 pandemic
Source: PLoS One. 2023 Jun 30;18(6):e0287730. doi: 10.1371/journal.pone.0287730 (PMC10313075; doi:10.1371/journal.pone.0287730)
Supplement: S1 Table — (DOCX) [file pone.0287730.s002.docx]

| **Supplementary Table 1.** Survey elements by core constructs |
| --- |
| **Demographics** |
| •   Neighborhood level: zip code, rural/urban |
| •   Individual level: age, gender, race/ethnicity, educational attainment, income, occupation, employment status, sexual orientation |
| **Perceived Susceptibility** |
| •   Perceived susceptibility to COVID-19 |
| •   Perceived susceptibility to preventable/early detected cancers |
| •   Perceived susceptibility to negative consequences associated with a cancer diagnosis |
| **Perceived Severity** |
| •   Health consequences of COVID-19 |
| •   Perceived stigma related to COVID-19 diagnosis |
| •   Perceived financial impacts of COVID-19 |
| **Perceived Barriers/Benefits** |
| •   Perceived stigma related to COVID-19 |
| •   Stress (including financial stressors), distress, discrimination/racism |
| •   Access to health information |
| •   Challenges related to social distancing (ability to get groceries, shortage of needed items, children home from school, ability to work from home, reduction of hours/pay, loss of employment, loss of health insurance) |
| •   Challenges related to the inability to engage in social distancing |
| •   Access to needed health services (not necessarily cancer-related) – ability to get medications/get acute care, access & use of telehealth/phone visits |
| •   Ability to obtain cancer care (clinic visits, get recommended treatments, get recommended surveillance tests/procedures, modifications to regimens, participation/access to clinical trials, pain control/management) |
| **Cancer Prevention and Control Behaviors** |
| •   Tobacco use (including vaping, e-cig)/Environmental tobacco exposure |
| •   Physical activity/inactivity |
| •   Fruit/vegetable intake |
| •   Alcohol intake |
| •   Breast, cervical, and colorectal screening |
| •   HPV vaccination |
| •   Adherence to treatment/surveillance |
