# Supplementary material for: The impact of socioeconomic status on changes in cancer prevention behavior during the COVID-19 pandemic
Source: PLoS One. 2023 Jun 30;18(6):e0287730. doi: 10.1371/journal.pone.0287730 (PMC10313075; doi:10.1371/journal.pone.0287730)
Supplement: S2 Table — * The aggregate score was used to classify participants into low socioeconomic status (SES scores 0 to 4), middle socioeconomic status (SES scores 5 and 6), or high socioeconomic status (SES scores 7 and 8). †Including unemployed, students, homemaker, or disabled. (DOCX) [file pone.0287730.s003.docx]

| **Supplementary Table 2**. Questions Used to Construct the Aggregate Socioeconomic Status Measure* | |
| --- | --- |
| **Socioeconomic Status Questions** | **Score** |
|  |  |
| **Household Income** |  |
| Thinking about members of your family living in your household, what is the combined annual income, meaning the total pre-tax income from all sources earned in the past year? |  |
| < $35,000 | 0 |
| $35,000 - $49,999 | 1 |
| $50,000 - $74,999 | 2 |
| $75,000 + | 3 |
| **Education** |  |
| What is the highest grade or level of school you completed? |  |
| High School or Less | 0 |
| Some College/Associate Degree | 1 |
| College degree | 2 |
| Graduate Degree | 3 |
| **Employment Status** |  |
| Which category best describes your occupational status in February 2020 prior to the stay-at-home orders put in place as a result of the COVID-19 Pandemic? |  |
| Employed Full- Part-Time | 2 |
| Retired | 1 |
| Other^†^ | 0 |
| * The aggregate score was used to classify participants into low socioeconomic status (SES scores 0 to 4), middle socioeconomic status (SES scores 5 and 6), or high socioeconomic status (SES scores 7 and 8). | |
| ^†^Including unemployed, students, homemaker, or disabled | |
