# Supplementary material for: The impact of socioeconomic status on changes in cancer prevention behavior during the COVID-19 pandemic
Source: PLoS One. 2023 Jun 30;18(6):e0287730. doi: 10.1371/journal.pone.0287730 (PMC10313075; doi:10.1371/journal.pone.0287730)
Supplement: S3 Table — (DOCX) [file pone.0287730.s004.docx]

| **Supplementary Table 3**. Definition of the cancer prevention behavior modification (CPBM) score | | | |  |
| --- | --- | --- | --- | --- |
| **Cancer Prevention Behavior** | **Adherence Status** | **Post-COVID-19 Modification*** | **Score Value** |  |
|  |  |  |  |  |
| **Physical Activity** | • Thinking about the last 30 days, in a typical week, how many days did you do any physical activity or exercise of at least moderate intensity, such as brisk walking, bicycling at a regular pace, and swimming at a regular pace? | • Have you changed the frequency of your physical activity compared to BEFORE the COVID-19 Pandemic? |  |  |
| **Recommendation Adherent** | Yes | More | 6 |  |
|  | Yes | Same | 5 |  |
|  | Yes | Less | 4 |  |
|  | No | More | 3 |  |
|  | No | Same | 2 |  |
|  | No | Less | 1 |  |
|  | Physically Inactive | None | 0 |  |
| **Fruit & Vegetable Intake** | • Now think about the foods you ate or drank during the past month, that is, the past 30 days, including meals and snacks. Not including juices, how often did you eat fruit? Include fresh, frozen or canned fruit. Do not include dry fruits. • During the past 30 days, how often did you eat vegetables other than potatoes? Include things like salad, cooked dried beans, corn, and broccoli. % | • Have you changed the amount of fruit and vegetables you consume per day compared to BEFORE the COVID-19 Pandemic? |  |  |
| **Recommendation Adherent** | Yes | More | 6 |  |
|  | Yes | Same | 5 |  |
|  | Yes | Less | 4 |  |
|  | No | More | 3 |  |
|  | No | Same | 2 |  |
|  | No | Less | 1 |  |
|  | No Daily Fruit or Vegetable Intake | None | 0 |  |
| **Alcohol Consumption** | • In the past 30 days, on how many days have you had a drink of an alcoholic beverage? • In the past 30 days, on how many days did you have 5 or more alcoholic drinks on the same occasion? | • Have you changed the amount of alcohol you drink compared to BEFORE the COVID-19 Pandemic? |  |  |
|  | Yes | More | 0 |  |
|  | Yes | Same | 1 |  |
|  | Yes | Less | 2 |  |
|  | No | More | 3 |  |
|  | No | Same | 4 |  |
|  | No | Less | 5 |  |
|  | No Alcohol Consumption | None | 6 |  |
| **Tobacco Use^†^** | • During the past 30 days, have you used any of the following tobacco or marijuana products? Select all that apply. | • Have you changed the frequency of tobacco or marijuana use compared to BEFORE the COVID-19 Pandemic? |  |  |
|  | Yes | More | 0 |  |
|  | Yes | Same | 2 |  |
|  | Yes | Less | 4 |  |
|  | No Tobacco Use | None | 6 |  |
| **Aggregate Cancer Prevention Score** | |  |  |  |
|  | Poor | | 0 - 13 |  |
|  | Average | | 14 - 15 |  |
|  | Good | | 16 -17 |  |
|  | Excellent | | 18+ |  |
| * Compared to Pre-COVID-19 Levels | | | |  |
| † a. Cigarettes, b. Little cigars, c. Cigarillos (e.g., Black & Mild), d. Hand-rolled cigarettes, e. Cigars (without marijuana), f. Blunts (with marijuana), g. Marijuana (rolled in a paper), h. Pipe, i. Bidi, j. Smokeless tobacco or dip, k. Electronic cigarettes containing nicotine, l. Electronic cigarettes containing marijuana, m. Hookah or waterpipe, n. Other (specify), o. I have not used any tobacco or marijuana products in the past 30 days – Go to the next section, p. Don’t know – Go to the next section, q. Prefer not to answer | | | |  |
