# Supplementary material for: The impact of socioeconomic status on changes in cancer prevention behavior during the COVID-19 pandemic
Source: PLoS One. 2023 Jun 30;18(6):e0287730. doi: 10.1371/journal.pone.0287730 (PMC10313075; doi:10.1371/journal.pone.0287730)
Supplement: S4 Table — Multinomial logistic regression models adjusting for age, sex, race-ethnicity, marital status, state, region of residence, health insurance, and socioeconomic status. * Included post-COVID-19 measures of physical activity, fruit and vegetable intake, alcohol consumption, and tobacco use compared to levels before the COVID-19 Pandemic. † Including participants who self-identified with more than one racial group. ‡ Included measures for education, household income, and occupational status. OR = Odds Ratio; CI = Confidence Interval. (DOCX) [file pone.0287730.s005.docx]

| **Supplementary Table 4.** Adjusted Odds Ratios for Factors Associated with Overall Cancer Prevention Behavior Changes Post the COVID-19 Pandemic (n=6,136) | | | |
| --- | --- | --- | --- |
| **Factor** | **Cancer Prevention Behavior Changes*** | | |
|  | **Poor vs. Excellent** | **Average vs. Excellent** | **Good vs. Excellent** |
|  | **OR (95% CI)** | **OR (95% CI)** | **OR (95% CI)** |
| **Age,** years |  |  |  |
| 18-34 | Ref. | Ref. | Ref. |
| 35-49 | 0.92 (0.68 - 1.24) | 0.83 (0.59 - 1.16) | 0.95 (0.66 - 1.37) |
| 50-64 | **0.61 (0.46 - 0.82)** | 0.79 (0.57 - 1.08) | 1.04 (0.74 - 1.47) |
| 65+ | **0.36 (0.25 - 0.50)** | 0.78 (0.54 - 1.13) | 0.98 (0.66 - 1.46) |
| **Sex** |  |  |  |
| Male | Ref. | Ref. | Ref. |
| Female | **0.64 (0.55 - 0.75)** | 0.87 (0.74 - 1.02) | 0.89 (0.75 - 1.05) |
| **Race Ethnicity** |  |  |  |
| White, non-Hispanic | Ref. | Ref. | Ref. |
| Black, non-Hispanic | 1.20 (0.86 - 1.68) | 1.36 (0.96 - 1.92) | 1.31 (0.91 - 1.89) |
| Hispanic | **0.58 (0.35 - 0.98)** | 1.23 (0.76 - 1.98) | 0.86 (0.49 - 1.48) |
| Other^†^ | **0.49 (0.35 - 0.69)** | 0.71 (0.50 - 1.00) | 0.57 (0.39 - 0.84) |
| **Marital status** |  |  |  |
| Single, Never Married | Ref. | Ref. | Ref. |
| Married/Living as Married | 1.05 (0.81 - 1.36) | 0.89 (0.68 - 1.17) | 1.23 (0.91 - 1.65) |
| Widowed, Separated or Divorced | 1.29 (0.95 - 1.74) | 1.07 (0.78 - 1.46) | 1.27 (0.90 - 1.79) |
| **Health Insurance** |  |  |  |
| Public & Private Insurance | Ref. | Ref. | Ref. |
| None | 0.64 (0.40 - 1.03) | 0.71 (0.44 - 1.14) | 0.58 (0.33 - 1.00) |
| Public Insurance | 1.18 (0.90 - 1.54) | 0.91 (0.69 - 1.20) | 0.91 (0.68 - 1.21) |
| Private Insurance | 0.90 (0.68 - 1.18) | 0.94 (0.71 - 1.24) | 0.96 (0.72 - 1.29) |
| **State** |  |  |  |
| Ohio | Ref. | Ref. | Ref. |
| Indiana | 0.94 (0.64 – 1.38) | 0.88 (0.59 – 1.30) | 0.94 (0.64 – 1.39) |
| **Region of Residence** |  |  |  |
| Metro | Ref. | Ref. | Ref. |
| Rural | 1.02 (0.87 - 1.20) | 1.14 (0.96 - 1.34) | 1.16 (0.98 - 1.37) |
| **Socioeconomic Status**^‡^ |  |  |  |
| High | Ref. | Ref. | Ref. |
| Middle | **1.40 (1.19 - 1.66)** | **1.24 (1.04 - 1.48)** | 1.15 (0.96 - 1.37) |
| Low | **1.55 (1.27 - 1.89)** | **1.50 (1.22 - 1.85)** | 1.14 (0.92 - 1.42) |
| Multinomial logistic regression models adjusting for age, sex, race-ethnicity, marital status, state, region of residence, health insurance, and socioeconomic status | | | |
| * Included post-COVID-19 measures of physical activity, fruit and vegetable intake, alcohol consumption, and tobacco use compared to levels before the COVID-19 Pandemic. | | | |
| † Including participants who self-identified with more than one racial group | | | |
| ‡ Included measures for education, household income, and occupational status | | | |
| OR= Odds Ratio; CI=Confidence Interval | | | |
