# Supplementary material for: The impact of socioeconomic status on changes in cancer prevention behavior during the COVID-19 pandemic
Source: PLoS One. 2023 Jun 30;18(6):e0287730. doi: 10.1371/journal.pone.0287730 (PMC10313075; doi:10.1371/journal.pone.0287730)
Supplement: S5 Table — All missing values were imputed using multiple imputations by chained equations to create ten imputed data sets. The parameter estimates obtained from each imputed data set were combined using the Rubin Method. * Passion regression with robust error variance with each 6-point cancer prevention behavior modification score as outcome adjusted for age, sex, race-ethnicity, marital status, region of residence, health insurance, and socioeconomic status. A higher score indicates better cancer prevention behavior modification post the COVID-19 Pandemic. † Including participants who self-identified with more than one racial group. ‡ Included measures for education, household income, and occupational status. RR = Relative Ratio; CI = Confidence Interval. (DOCX) [file pone.0287730.s006.docx]

| **Supplementary Table 5.** Adjusted Relative Ratios (aRR) for Factors Associated with Higher* Cancer Prevention Behavior Modification Score Post the COVID-19 Pandemic Using Multiple Imputations (n=9,280) | | | | |
| --- | --- | --- | --- | --- |
| **Factor** | **More Physical Activity** | **More Fruit & Vegetable Intake** | **Less Alcohol Consumption** | **Less Tobacco Use** |
|  | **aRR (95% CI)** | **aRR (95% CI)** | **aRR (95% CI)** | **aRR (95% CI)** |
| **Age,** years |  |  |  |  |
| 18-34 | Ref. | Ref. | Ref. | Ref. |
| 35-49 | 0.94 (0.88 - 1.01) | 0.97 (0.92 - 1.02) | **1.09 (1.03 - 1.14)** | 1.01 (0.98 - 1.05) |
| 50-64 | 0.96 (0.90 - 1.03) | 0.98 (0.93 - 1.03) | **1.20 (1.15 - 1.26)** | **1.06 (1.03 - 1.10)** |
| 65+ | 0.99 (0.91 - 1.08) | 1.02 (0.96 - 1.08) | **1.26 (1.19 - 1.32)** | **1.17 (1.13 - 1.21)** |
| **Sex** |  |  |  |  |
| Male | Ref. | Ref. | Ref. | Ref. |
| Female | **0.96 (0.93 - 0.99)** | **1.10 (1.08 - 1.13)** | **1.07 (1.05 - 1.09)** | **1.07 (1.05 - 1.08)** |
| **Race Ethnicity** |  |  |  |  |
| White, non-Hispanic | Ref. | Ref. | Ref. | Ref. |
| Black, non-Hispanic | **0.85 (0.79 - 0.93)** | **1.05 (1.00 - 1.11)** | 1.03 (0.99 - 1.07) | 1.02 (0.99 - 1.05) |
| Hispanic | 1.01 (0.90 - 1.12) | 0.99 (0.90 - 1.09) | 0.97 (0.90 - 1.04) | 0.97 (0.92 - 1.02) |
| Other^†^ | 1.03 (0.96 - 1.11) | **1.08 (1.01 - 1.15)** | 1.13 (1.08 - 1.18) | **1.03 (1.00 - 1.06)** |
| **Marital status** |  |  |  |  |
| Single, Never Married | Ref. | Ref. | Ref. | Ref. |
| Married/Living as Married | 1.00 (0.94 - 1.06) | 1.01 (0.96 - 1.05) | 0.98 (0.95 - 1.01) | 1.01 (0.99 - 1.04) |
| Widowed, Separated or Divorced | 0.97 (0.91 - 1.04) | 0.96 (0.91 - 1.01) | 0.97 (0.94 - 1.00) | 0.98 (0.95 - 1.01) |
| **Health Insurance** |  |  |  |  |
| Public & Private Insurance | Ref. | Ref. | Ref. | Ref. |
| None | 1.04 (0.92 - 1.18) | **0.89 (0.81 - 0.98)** | 1.04 (0.97 - 1.11) | **0.89 (0.84 - 0.94)** |
| Public Insurance | 1.00 (0.94 - 1.07) | 0.97 (0.93 - 1.02) | 1.01 (0.99 - 1.04) | **0.96 (0.94 - 0.99)** |
| Private Insurance | **1.08 (1.01 - 1.16)** | 1.03 (0.98 - 1.07) | 0.94 (0.92 - 0.97) | **1.06 (1.03 - 1.08)** |
| **State** |  |  |  |  |
| Ohio | Ref. | Ref. | Ref. | Ref. |
| Indiana | 0.99 (0.90 - 1.09) | 0.97 (0.91 - 1.04) | 1.01 (0.97 - 1.05) | 1.01 (0.99 - 1.03) |
| **Region of Residence** |  |  |  |  |
| Metro | Ref. | Ref. | Ref. | Ref. |
| Rural | 1.00 (0.97 - 1.04) | 0.98 (0.95 - 1.00) | **1.03 (1.01 - 1.04)** | 0.99 (0.98 - 1.00) |
| **Socioeconomic Status^‡^** |  |  |  |  |
| High | Ref. | Ref. | Ref. | Ref. |
| Middle | **0.89 (0.86 - 0.92)** | **0.92 (0.89 - 0.95)** | **1.06 (1.04 - 1.08)** | **0.98 (0.97 - 0.99)** |
| Low | **0.76 (0.73 - 0.80)** | **0.88 (0.85 - 0.91)** | **1.16 (1.13 - 1.19)** | **0.94 (0.92 - 0.95)** |
| All missing values were imputed using multiple imputations by chained equations to create ten imputed data sets. The parameter estimates obtained from each imputed data set were combined using the Rubin Method. | | | | |
| * Passion regression with robust error variance with each 6-point cancer prevention behavior modification score as outcome adjusted for age, sex, race-ethnicity, marital status, region of residence, health insurance, and socioeconomic status. A higher score indicates better cancer prevention behavior modification post the COVID-19 Pandemic | | | | |
| † Including participants who self-identified with more than one racial group | | | | |
| ‡ Included measures for education, household income, and occupational status | | | | |
| RR=Relative Ratio; CI=Confidence Interval | | | | |
