# Supplementary material for: The impact of socioeconomic status on changes in cancer prevention behavior during the COVID-19 pandemic
Source: PLoS One. 2023 Jun 30;18(6):e0287730. doi: 10.1371/journal.pone.0287730 (PMC10313075; doi:10.1371/journal.pone.0287730)
Supplement: S6 Table — All missing values were imputed using multiple imputations by chained equations to create ten imputed data sets. The parameter estimates obtained from each imputed data set were combined using the Rubin Method. Multinomial logistic regression models adjusting for age, sex, race-ethnicity, marital status, region of residence, health insurance, and socioeconomic status. * Include post-COVID-19 measures of physical activity, fruit and vegetable intake, alcohol consumption, and tobacco use compared to levels before the COVID-19 Pandemic. † Including participants who self-identified with more than one racial group. ‡ Included measures for education, household income, and occupational status. OR = Odds Ratio; CI = Confidence Interval. (DOCX) [file pone.0287730.s007.docx]

| **Supplementary Table 6.** Adjusted Odds Ratios for Factors Associated with Overall Cancer Prevention Behavior Modifications Post the COVID-19 Pandemic Using Multiple Imputations (n=9,280) | | | |
| --- | --- | --- | --- |
| **Factor** | **Cancer Prevention Behavior Modifications*** | | |
|  | **Poor vs. Excellent** | **Average vs. Excellent** | **Good vs. Excellent** |
|  | **OR (95% CI)** | **OR (95% CI)** | **OR (95% CI)** |
| **Age,** years |  |  |  |
| 18-34 | Ref. | Ref. | Ref. |
| 35-49 | 0.91 (0.70 - 1.18) | 0.85 (0.63 - 1.14) | 0.92 (0.66 - 1.26) |
| 50-64 | **0.61 (0.47 - 0.79)** | 0.82 (0.62 - 1.07) | 1.07 (0.79 - 1.44) |
| 65+ | **0.36 (0.27 - 0.48)** | 0.74 (0.53 - 1.01) | 1.00 (0.70 - 1.42) |
| **Sex** |  |  |  |
| Male | Ref. | Ref. | Ref. |
| Female | **0.64 (0.56 - 0.73)** | **0.86 (0.75 - 1.00)** | **0.85 (0.73 - 1.00)** |
| **Race Ethnicity** |  |  |  |
| White, non-Hispanic | Ref. | Ref. | Ref. |
| Black, non-Hispanic | 1.16 (0.86 - 1.55) | 1.21 (0.91 - 1.62) | 1.15 (0.82 - 1.61) |
| Hispanic | 1.02 (0.68 - 1.54) | 1.15 (0.73 - 1.82) | 1.01 (0.64 - 1.61) |
| Other^†^ | **0.50 (0.37 - 0.66)** | **0.71 (0.52 - 0.97)** | **0.60 (0.42 - 0.85)** |
| **Marital status** |  |  |  |
| Single, Never Married | Ref. | Ref. | Ref. |
| Married/Living as Married | 0.99 (0.79 - 1.23) | 0.91 (0.73 - 1.14) | 1.08 (0.85 - 1.39) |
| Widowed, Separated or Divorced | 1.28 (1.00 - 1.64) | 1.10 (0.85 - 1.41) | 1.17 (0.88 - 1.57) |
| **Health Insurance** |  |  |  |
| Public & Private Insurance | Ref. | Ref. | Ref. |
| None | 1.08 (0.72 - 1.62) | 0.81 (0.53 - 1.22) | 0.71 (0.44 - 1.14) |
| Public Insurance | 1.01 (0.81 - 1.26) | 0.89 (0.70 - 1.13) | 0.88 (0.70 - 1.11) |
| Private Insurance | 0.86 (0.68 - 1.10) | 0.89 (0.69 - 1.14) | 0.97 (0.76 - 1.24) |
| **State** |  |  |  |
| Ohio | Ref. | Ref. | Ref. |
| Indiana | 0.97 (0.69 - 1.35) | 0.84 (0.59 - 1.19) | 0.87 (0.62 - 1.23) |
| **Region of Residence** |  |  |  |
| Metro | Ref. | Ref. | Ref. |
| Rural | 1.00 (0.87 - 1.15) | 1.08 (0.94 - 1.25) | 1.11 (0.97 - 1.28) |
| **Socioeconomic Status**^‡^ |  |  |  |
| High | Ref. | Ref. | Ref. |
| Middle | **1.32 (1.14 - 1.54)** | **1.21 (1.03 - 1.41)** | 1.13 (0.96 - 1.33) |
| Low | **1.54 (1.29 - 1.83)** | **1.44 (1.20 - 1.72)** | 1.11 (0.92 - 1.35) |
| All missing values were imputed using multiple imputations by chained equations to create ten imputed data sets. The parameter estimates obtained from each imputed data set were combined using the Rubin Method. | | | |
| Multinomial logistic regression models adjusting for age, sex, race-ethnicity, marital status, region of residence, health insurance, and socioeconomic status | | | |
| * Include post-COVID-19 measures of physical activity, fruit and vegetable intake, alcohol consumption, and tobacco use compared to levels before the COVID-19 Pandemic. | | | |
| † Including participants who self-identified with more than one racial group | | | |
| ‡ Included measures for education, household income, and occupational status | | | |
| OR= Odds Ratio; CI=Confidence Interval | | | |
